# Droplet-Based Microfluidics in Single-Bacterium Analysis: Advancements in Cultivation, Detection, and Application

**DOI:** 10.3390/bios15080535

**Published:** 2025-08-15

**Authors:** Haiyan Ma, Yuewen Zhang, Ren Shen, Yanwei Jia

**Affiliations:** 1 College of Chemistry and Chemical Engineering, Lanzhou University, Lanzhou 730000, China; mahy2021@lzu.edu.cn; 2 State Key Laboratory of Analog and Mixed-Signal VLSI, Institute of Microelectronics, University of Macau, Macau SAR 999078, China; yanweijia@um.edu.mo; 3 Faculty of Science and Technology, University of Macau, Macau SAR 999078, China; 4 MoE Frontiers Science Center for Precision Oncology, University of Macau, Macau SAR 999078, China

**Keywords:** droplet-based microfluidics, single-cell analysis, bacteria, uncultured microorganisms

## Abstract

Microorganisms exhibit remarkable diversity, making their comprehensive characterization essential for understanding ecosystem functioning and safeguarding human health. However, traditional culture-based methods entail inherent limitations for resolving microbial heterogeneity, isolating slow-growing microorganisms, and accessing uncultivated microbes. Conversely, droplet-based microfluidics enables a high-throughput and precise platform for single-bacterium manipulation by physically isolating individual cells within microdroplets. This technology presents a transformative approach to overcoming the constraints of conventional techniques. This review outlines the fundamental principles, recent research advances, and key application domains of droplet-based microfluidics, with a particular focus on innovations in single-bacterium encapsulation, sorting, cultivation, and functional analysis. Applications such as antibiotic susceptibility testing, enzyme-directed evolution screening, microbial interaction studies, and the cultivation of novel bacterial species are discussed, underscoring the technology’s broad potential in microbiological research and biotechnology.

## 1. Introduction

Microorganisms have shaped our planet and its inhabitants for over 3.5 billion years [1]. Microorganisms play a key role in geochemistry and ecosystems, significantly affecting human life, both past and future [2]. Bacteria, as an important subset of microorganisms, are widely distributed in the deep sea, polar regions, soil, and plants and animals [3]. A full comprehension of bacteria is of significant importance to a wide range of fields, including human health, medicine, and environmental science. Within the human body, a normal microbiota, such as intestinal bacteria, plays a crucial role in maintaining human health. In particular, these microorganisms facilitate digestion, enhance immune function, produce beneficial compounds, and defend against pathogenic bacteria [4]. In the medical field, bacterial analysis allows for the accurate identification of strain-specific antimicrobial resistance profiles [5]. This methodology enables the detailed investigation of bacterial pathogenesis, consequently accelerating both the discovery of novel therapeutic agents and the advancement of precision medicine. In environmental science, bacterial analysis monitors and assesses microbial communities, an essential process for understanding ecosystem homeostasis and material cycling [6].

Due to the microscopic size and compositional complexity of bacteria in their native environments, bacterial studies often require primary culture, isolation, and pure culture for subsequent analyses [7]. Nevertheless, conventional culture methodologies based on solid or liquid media exhibit inherent constraints in bacterial analysis. Firstly, traditional plate culture methods rely predominantly on colony morphology and population-level metabolic profiling [8] yet fail to differentiate intercellular genetic and phenotypic variations, thereby potentially disregarding microbial heterogeneity and obscuring crucial biological insights. Heterogeneity refers to inherent biological variation between individual bacterial cells (e.g., in growth rate, gene expression, and metabolic activity). Secondly, during conventional colony selection procedures, fast-growing species predominate on cultivation media by outcompeting their slow-growing counterparts [9], consequently complicating the isolation of slow-growing bacterial populations and necessitating labor-intensive reiterative purification protocols. Thirdly, over 99% of environmental microorganisms remain unculturable through conventional cultivation methods, a phenomenon termed microbial “dark matter”, the study of which is imperative for advancing microbiological research [10]. To overcome these limitations of the conventional approaches, there is a critical requirement for novel technological solutions capable of achieving accurate, rapid, and high-throughput bacterial isolation, cultivation, and analytical processes.

The emergence of microfluidics offers a promising approach to the aforementioned challenges of bacteria analysis. Microfluidics represents a technology that enables the precise manipulation of fluids at the microscale, integrating diverse laboratory operations—including sample processing, reactions, and assays onto a single chip. This integration offers significant advantages, including a high-throughput, low reagent consumption, and integration [11,12]. As a significant branch of microfluidic technology, droplet-based microfluidics has demonstrated substantial advantages in microbiology. This method precisely encapsulates individual bacterial cells within discrete droplets, utilizing the oil–phase interface to eliminate interspecies competition and environmental interference, thus enabling bacterial functional analysis at the single-cell level, a level of analysis essential to elucidating microbial heterogeneity and functional diversity [13]. Droplet-based microfluidics further enhances system automation and portability by enabling the encapsulation, isolation, incubation, and analysis of individual bacteria within droplets. Moreover, the generation rates of thousands to tens of thousands of droplets per second facilitate the parallel analysis of millions of single cells within short timeframes [14]. Owing to these inherent advantages, this technology has found broad application across multiple biological domains, including single-cell sequencing [15], microbial culture and detection [16,17,18], and drug discovery [19].

**Scheme 1:**
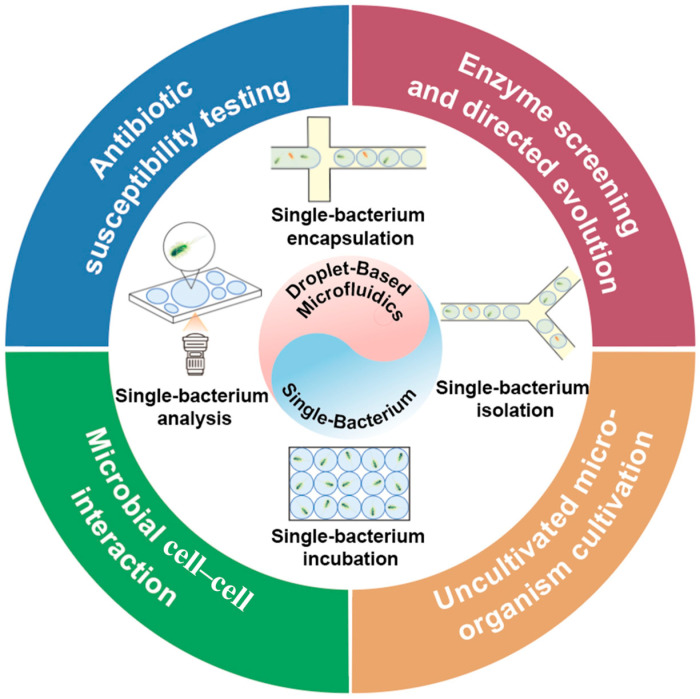
Applications of droplet-based microfluidics in single-bacterium research, with an emphasis on fundamental techniques and their diverse applications.

In this review, we focus on the core methods and applications of droplet-based microfluidics for the single-cell analysis of bacteria (Scheme 1). First, we introduce the fundamental principles and innovations across droplet-based microfluidic platforms for single-bacterium analysis, including encapsulation, isolation, culture, and monitoring. Subsequently, we highlight key application domains in single-bacterium research, featuring representative applications in antibiotic susceptibility testing (AST), enzyme-directed evolution, microbial interaction studies, and the isolation of uncultivated microorganisms.

## 2. Principles of Droplet-Based Microfluidics for Single-Bacterium Analysis

Droplet-based microfluidics focuses on generating and manipulating discrete microdroplets, with volumes ranging from nanoliters to femtoliters, using immiscible fluid systems. This method offers four key advantages: (1) Single-cell isolation: Individual bacterial cells can be encapsulated in separate droplets, eliminating competition from fast-growing strains that often suppress slower populations on agar plates [20]. This isolation enables true single-cell assays and minimizes population-level interference [21]. (2) Native microenvironment emulation: Temperature [22], oxygen concentration [23], nutrient gradients, and other parameters can be dynamically tuned within each droplet, allowing researchers to recreate otherwise-inaccessible microhabitats and cultivate species deemed “unculturable,” such as those from soil or deep sea environments. (3) High-throughput operation: Rapid droplet generation supports the parallel cultivation of numerous single bacterial cells within reduced timeframes. (4) Integrated workflows: Encapsulation, cultivation, and detection can be combined on a single microfluidic device, streamlining experimental workflows and enabling automated, highly reproducible microbiological assays.

A typical droplet-based microfluidic platform consists of several functional modules, including droplet generation, splitting, merging, sorting, and incubation. Table 1 summarizes the underlying techniques and representative applications of each module. Depending on the specific application requirements, these manipulations can be used individually or integrated into a single chip, affording programmable control over droplet size, morphology, and chemical composition. The following sections highlight droplet generation and manipulation strategies and discuss their specific applications in single-bacterium studies.

### 2.1. Encapsulation of a Single Bacterium Using Droplet-Based Microfluidics

Droplet-based microfluidic technology represents a transformative platform for microbiological analysis. By generating monodisperse droplets, it enables precise single-cell encapsulation, allowing each bacterium to grow independently at ultrahigh cell densities within these compartmentalized systems [38]. The encapsulation of single bacteria in droplets is achieved through the precise manipulation of fluid dynamics in microfluidic systems. When a bacterial suspension flows through the droplet generation chip, the aqueous phase containing bacteria is sheared by the oil phase into discrete microdroplets. During this process, bacteria are randomly distributed into droplets along with the reagent, following the Poisson distribution law. Based on the developmental stages, droplet generation methods can be categorized as traditional (passive hydrodynamic) and emerging (actively controlled) categories. Traditional approaches are grounded in passive hydrodynamic principles, relying on the interplay between continuous and dispersed phases for droplet formation [39]. In this passive regime, bacterial encapsulation inherently follows Poisson statistics. While single-bacterium encapsulation occurs stochastically, its efficiency is constrained, typically reaching maximum levels of 30–40% under optimized conditions (e.g., low cell concentration and small droplet volume) [8]. This limitation results in a high proportion of empty droplets and droplets containing multiple cells, which complicates downstream single-cell analysis and is a major bottleneck for applications requiring high purity and throughput. In contrast, emerging methodologies overcome the constraints of stochastic encapsulation via structural innovations or actively controlled strategies, contributing to enhanced efficiency and precision in single-bacterium encapsulation.

#### 2.1.1. Traditional Droplet Generation Methods

**Figure 1:**
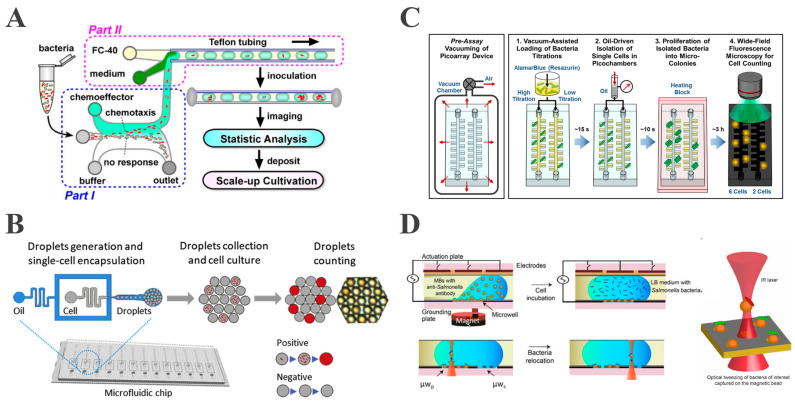
Encapsulation of a single bacterium using droplet-based microfluidics. (**A**) Schematic of the device for droplet generation in T-junction (reproduced with permission from [41], copyright 2016, Springer Nature). (**B**) Schematic of detection of single *Salmonella* in flow-focusing microchannels (reproduced with permission from [44], copyright 2020, Elsevier). (**C**) Overview of picoarray for counting of viable bacteria (reproduced with permission from [49], copyright 2018, American Chemical Society). (**D**) Schematic of using the OTs-integrated EWOD platform for capturing single bacterium (reproduced with permission from [50], copyright 2020, MDPI).

Traditional droplet generation includes three main methods: T-junction, flow-focusing, and co-flow-focusing. The T-junction structure was first proposed by Thorsen et al. In this method, the dispersed phase flows perpendicularly into the continuous phase stream, where shear forces create uniform droplets [40]. Dong et al. mixed a bacterial suspension with growth medium in a T-junction, generating nanoscale droplets that encapsulated a single *Escherichia coli* (*E. coli*) (Figure 1A). Single-cell encapsulation was accomplished by adjusting initial *E. coli* concentrations according to Poisson’s distribution statistics, with approximately 10% of the droplets containing a single bacterium [41].

Flow-focusing, first proposed by Anna [42] and Dreyfus [43] et al., forms droplets at cross-structures by squeezing the continuous phase fluid from both sides, yielding more stable and uniform droplets than a single-sided T-junction. An et al. employed flow-focusing microchannels to co-encapsulate fluorescent dyes with single *Salmonella* within homogeneous picoliter droplets [44] (Figure 1B), achieving pathogen detection within 5 h and a detection limit of 50 CFU/mL.

The co-flow-focusing structure was originally developed by Weitz et al. for generating monodisperse emulsions [45]. The co-flowing combines two microchannels in a coaxial nested configuration, where the continuous phase surrounds the dispersed phase concentrically [46]. Terekhov et al. employed a co-flow microfluidic device to construct a water-in-oil emulsion for encapsulating individual bacterial cells within monodisperse droplets, thereby forming a microculture environment capable of maintaining bacterial viability [47]. This technology enables the screening of oral microbiota for bacterial species that inhibit *Staphylococcus aureus* (*S. aureus*) growth, followed by the identification of inhibitory genera via 16S rRNA sequencing and secretome analysis. However, co-flow-focusing devices, which are primarily fabricated using micron-scale capillaries, demonstrate comparatively low reproducibility, and the coaxial flow configuration often produces polydisperse droplets [48].

#### 2.1.2. Emerging Droplet Generation Techniques

While the abovementioned conventional methods permit efficient single-droplet generation, they lack precise individual droplet manipulation capabilities and are inadequate for specialized experimental requirements, including real-time single-bacterium monitoring. This is primarily because the single-bacterium encapsulation process is governed by Poisson’s statistics, resulting in suboptimal encapsulation efficiency. Notably, emerging droplet generation strategies, including microarray, electrowetting-on-dielectric (EWOD) systems, and pneumatic microvalves, demonstrate notable improvements in droplet manipulation precision, single-bacterium encapsulation efficiency, and enhanced versatility.

Recently, Hsieh et al. developed a vacuum-assisted sample loading system with oil-driven digitization that stochastically confines single-cell bacteria in picoarray isolation chambers [49]. This approach, integrated with fluorescent dye-based amplification detection, enables precise live cell quantification within 3 h (Figure 1C). In particular, the microarray architecture facilitates physical isolation of individual bacterial cells and enhances assay precision. Nevertheless, droplet manipulation using this approach is primarily performed at a static level, thereby limiting its dynamic manipulation capabilities.

Meanwhile, EWOD technology enables active droplet generation through the precise control of microdroplets via surface wetting forces, by utilizing electrode arrays functionalized with hydrophobic dielectric coatings. The programmable electrode actuation allows the droplets to be transported, merged, mixed, and split along arbitrary paths through a controlled electrode array [51]. Extending this technology, Kumar integrated EWOD digital microfluidics with optical tweezers (OTs) to capture a single bacterium using functionalized magnetic beads [50]. The OTs enable selective capture while providing continuous cell imaging for dynamic behavior assessment (Figure 1D). This method overcomes traditional Poisson’s distribution limitations by enabling the active selection of a targeted single bacterium according to fluorescence expression, achieving deterministic encapsulation and manipulation. This system additionally provides dynamic real-time imaging and monitoring of individual bacteria, thus permitting the direct investigation of their behavioral dynamics. Although the optical tweezer methodology can improve encapsulation efficiency, its throughput is highly limited.

Beyond conventional approaches, Jeong et al. developed an addressable static droplet array (SDA) integrated with double-layer pneumatic microvalves [52]. This system enables the digital control of individual pico-droplets through selective hydrodynamic trapping, releasing, and rearrangement. Crucially, the initial random encapsulation of bacteria followed a Poisson’s distribution, with only 45.8% of droplets containing single cells. By repetitive selective trapping and releasing operations, the SDA actively discarded droplets with zero or multiple bacteria, thereby overcoming the Poisson distribution limitations. The final array achieved 100% single-bacterium encapsulation efficiency, demonstrating the precise isolation and retrieval of target cells for downstream analysis.

### 2.2. Isolation of a Single Bacterium via Droplet Sorting Techniques

Droplet sorting denotes the selective isolation of specific droplets from populations for targeted processing and analytical procedures. Droplet sorting technology facilitates the rapid and high-throughput isolation of a targeted single bacterium from complex microbial consortia, enabling subsequent analytical procedures. Droplet sorting is classified into two distinct operational modalities: passive and active sorting mechanisms.

#### 2.2.1. Passive Droplet Sorting

**Figure 2:**
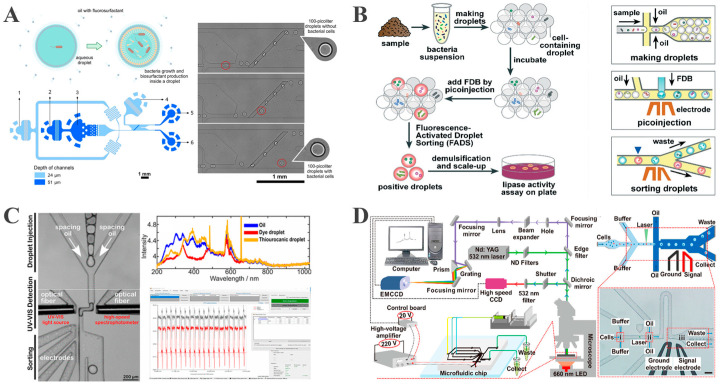
Isolation of a single bacterium based on the droplet sorting technique. (**A**) A microfluidic platform for size-based passive sorting of droplets exploiting interfacial tension reduction (reproduced with permission from [55], copyright 2024, Royal Society of Chemistry). (**B**) Schematic representation of the isolation of targeted single bacterial droplets using FADS (reproduced with permission from [56], copyright 2018, Royal Society of Chemistry). (**C**) High-throughput label-free identification of chemicals in droplets and on-demand collection through UVADS (reproduced with permission from [57], copyright 2021, American Chemical Society). (**D**) RADS platform for label-free high-throughput screening of unicellular microalgae (reproduced with permission from [58], copyright 2017, American Chemical Society).

Passive sorting utilizes microchannel structural designs and droplet physical properties to enable sorting processes without external energy input. The most widely used approaches are deterministic lateral displacement (DLD) [53] and inertial sorting techniques [54]. Recently, Staskiewicz et al. developed a passive droplet sorting system based on interfacial tension, exploiting surfactant-producing bacteria-induced interfacial tension changes to achieve high-throughput single-bacterium isolation [55]. This methodology can encapsulate a single cell within 100-picoliter droplets for clonal expansion while implementing passive sorting through interfacial tension gradients at operational frequencies reaching 250 droplets per second, achieving an enrichment factor of up to 4250 for the surfactant-producing *Bacillus* sp. ANT_WA51 (Figure 2A). This approach overcomes the throughput limitations of conventional microbial screening while significantly enhancing the isolation efficiency for rare functional strains. However, passive sorting systems exhibit limited flexibility due to the inherent coupling between sorting bias and predefined parameters, restricting dynamic adaptation in response to experimental requirements [6]. In contrast, active sorting systems utilizing an external field (electric, acoustic, or optical) demonstrate enhanced suitability for precision droplet manipulation under complex conditions.

#### 2.2.2. Active Droplet Sorting

Dielectrophoresis (DEP) is the most widely used active sorting method, combining a high throughput with precise addressability [59]. In a non-uniform alternating electric field, droplets become polarized and experience a directional force; they migrate toward regions of high field strength (positive DEP) or low field strength (negative DEP), depending on their dielectric properties [6]. DEP must be combined with upstream detection technologies to construct the operational sequence of “droplet property identification to droplet sorting execution.” Through fluorescence detection, ultraviolet absorption, and other technologies, this system achieves real-time measurements of droplet fluorescence intensity, absorbance, and related physicochemical parameters. Following droplet identification, DEP classifies targeted droplets in real time.

Qiao et al. developed a high-throughput screening platform based on fluorescence-activated droplet sorting (FADS) to overcome the low-throughput limitation of conventional plate screening methods for lipolytic strain selection [56]. This platform employs droplet microfluidics to encapsulate single bacterial cells within monodisperse droplets, followed by the incubation and subsequent infusion of a fluorogenic substrate (Figure 2B). Lipase-secreting strains amplify fluorescence through enzymatic substrate hydrolysis, which is detected by an optical system. By applying an electrical field, high-fluorescence droplets are sorted via positive DEP at 2 × 10^6^ droplets per hour. Capitalizing on this high-throughput capacity, this method identified 47 lipolytic strains from extreme environments (Tibetan hot springs, oil-polluted soils), offering a new approach for enzyme discovery. However, the FADS platform requires fluorescent labeling, and because of this, the non-enzymatic hydrolysis of substrates could potentially interfere with signal specificity. Furthermore, single-wavelength detection in FADS compromises the analytical flexibility of complex sample matrices.

To address the limitations of fluorescence-based platforms, Duncombe et al. developed UV–visible spectroscopy-activated droplet sorting (UVADS), a label-free system that leverages full-spectrum absorbance detection. This platform captures 2100 spectra per second using integrated SpectraSorter software, enabling the real-time compositional analysis of individual microdroplets [57]. Following incubation, bacterial growth leads to increased absorbance at 280 nm, along with changes in OD600, allowing the DEP-based redirection of target droplets into collection channels (Figure 2C). In contrast to FADS, UVADS can aid in monitoring single-bacterium growth without requiring fluorescent probes. Therefore, UVADS is expanding both analytical dimensions and application scope, particularly for the label-free analysis of complex biological specimens.

In addition to UV–visible detection, Raman spectroscopy is also widely employed as a label-free technique with strong potential for biological sample analysis. Wang et al. designed a Raman-activated droplet sorting (RADS) platform integrating single-cell Raman profiling with microfluidic droplet generation and DEP-based sorting [58]. In this workflow, cells are first analyzed via Raman spectroscopy before encapsulation; target microalgae producing astaxanthin are then sorted using DEP at a throughput of 260 cells per minute and 98.3% sorting accuracy (Figure 2D). This system enables the effective enrichment of functional microalgal strains from heterogeneous populations, offering a powerful platform for high-throughput phenotyping and single-cell omics in microalgal biotechnology.

### 2.3. Single-Bacterial Cultivation and Analysis in Droplet-Based Microfluidics

Microbial culture remains essential for studying microorganisms, but conventional methods like agar plating and nutrient broth are limited by susceptibility to cross-contamination, low throughput, and the masking of single-cell heterogeneity due to population effects [60]. In turn, droplet-based microfluidics provides a novel approach to microbial culture by encapsulating a single bacterium in picoliter-to-nanoliter droplets to form individual microreactors, thus avoiding population competition and contamination while enabling the high-throughput generation of thousands of single-cell droplets per second and real-time monitoring.

Current microbial culture methods based on droplet microfluidics are categorized into off-chip and on-chip approaches. Off-chip culture necessitates transferring encapsulated single-bacterial droplets to a Petri dish for incubation, and this transfer process risks causing droplet rupture or fusion, which would compromise the microbial growth conditions within the droplets. On the other hand, on-chip single-bacterial culture integrates modules for droplet encapsulation, single-bacterial cultivation, and detection, enabling the dynamic in situ regulation of temperature, gas composition, and other environmental parameters, as well as the real-time monitoring of single-bacterial growth via fluorescence and Raman spectroscopy, among other techniques. These capabilities establish on-chip culture as a highly advantageous approach for microbial studies.

#### 2.3.1. Construction of a Single-Bacterial Culture Microenvironment

The droplet microenvironment refers to the local environment constructed by microscopic droplets ranging from picoliters to nanoliters in microfluidic systems and exhibiting precisely controllable physical and chemical properties. These droplets form independent, sealed microreactors enabling the precise regulation of internal environmental parameters, such as nutrient concentration, temperature, pH, and osmotic pressure [8,61]. Maintaining a stable microenvironment within chips is crucial for accurate bacterial cultivation, as even minor environmental fluctuations can compromise growth and reproducibility. However, current droplet-based microfluidic single-bacterial culture faces two key challenges: inefficient large-scale droplet array generation, which restricts the throughput needed for functional screening, and rapid droplet evaporation, due to the small volume of picoliter-scale compartments, hindering long-term monitoring [62].

**Figure 3:**
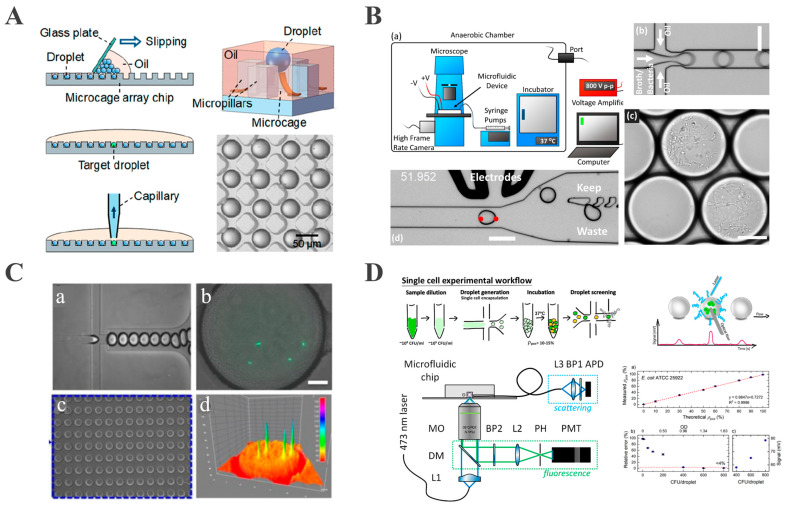
Droplet-based microbial cultivation and analysis. (**A**) A large-scale 2D monolayer droplet array for single-cell culture (reproduced with permission from [63], copyright 2019, American Chemical Society). (**B**) A droplet-based microfluidic system for efficient isolation and cultivation of enteric anaerobes (reproduced with permission from [70], copyright 2020, eLife). (**C**) Integrated microfluidic platform for rapid bacterial growth analysis via fluorescence imaging (reproduced with permission from [71], copyright 2017, Springer Nature). (**D**) A platform for monitoring bacterial cell density in droplets using label-free scattered light techniques (reproduced with permission from [72], copyright 2020, American Chemical Society).

To overcome the inefficiency of conventional droplet array generation, Xu et al. developed a microcage array chip-based droplet culture system. Through rapid oil–phase diffusion via microcage–microcolumn interstices, a two-dimensional array comprising approximately 10^6^ picoliter droplets was fabricated on a 5.5 × 5.5 cm chip within 90 s (Figure 3A). The precise droplet positioning and a microfluidic robot with 100% retrieval efficiency enabled the screening of thermophilic esterase AFEST-expressing bacteria. Their system also allowed single-bacterium encapsulation and fluorescence-based real-time monitoring at 37 °C [63]. However, this approach lacked explicit evaporation control, making it unsuitable for extended culture periods.

Droplet evaporation presents a significant challenge in long-term microbial cultivation. The primary strategies to minimize evaporation involve establishing a sealed environment with gas-impermeable materials or coating the droplet surface with silicone oil to create a protective layer. To avoid the droplet shrinkage issue caused by evaporation, Li et al. used gas-impermeable Teflon tubing as the culture carrier, with sealed ends forming an airtight environment to significantly suppress evaporation—after 24h incubation at 37 °C, droplet size changed by less than 2%. With that, they implemented a sealed environment for long-term bacteria cultivation in their nanodroplet platform and applied this platform to screen antibiotic combinations. The resazurin dye was used to monitor the real-time bacterial activity over a 24 h period, verifying the feasibility of long-term bacterial culture in droplets [64].

To suppress evaporation, Wu et al. immersed the generated droplet arrays in Fluorinert FC40 oil, creating a sealed environment. This oil coverage effectively prevented evaporation, allowing droplet storage at room temperature for over 48 h with less than 35% volume loss. Leveraging this stability, the platform enabled the successful encapsulation and 72 h dynamic tracking of division and morphology in single *E. coli* cells. [65]. While sealed environments can ensure minimum droplet evaporation during bacteria culture and reduce volume shrinkage, gas exchange may be blocked in these setups, affecting cell viability (especially, of aerobic bacteria species) in long-term cultivation. To solve this problem, Li et al. adopted an anti-evaporation chip structure on a digital microfluidic chip [62]. A sealed square spacer was placed between the top and bottom plates of the DMF chip. Silicone oil was used, but the oil only existed in the gaps between the spacer and the bottom/top plates. This formed an enclosed environment to prevent droplet evaporation, while the oil-free internal chamber allowed for gas exchange between the bacteria-containing droplets and the environment. With this setup, droplets showed no significant evaporation during the incubation process.

Apart from sealing the culturing chambers, mitigating environmental humidity is another solution to avoid droplet evaporation during bacterial culture. To mitigate evaporation through humidity control, Cui et al. incorporated a water jacket layer positioned above the culture chambers to maintain a near-saturated vapor environment. Numerical simulation confirmed negligible humidity gradients around the chambers, extending the evaporation time beyond 20 h. This controlled humidity enabled successful 5–7 day long-term culture and the growth monitoring of diverse oral bacteria (*Streptococcus mutans*, *Actinomyces viscosus*, and *Fusobacterium nucleatum*) under varying oxygen concentrations [66].

The long-term cultivation of bacteria inside droplets also has detrimental effects on cell viability. The side effects can be attributed to several aspects, including nutrient depletion, waste accumulation, limited oxygen exchange, drastic pH fluctuation, and confinement stress in tiny droplets. These challenges can be partially resolved with optimized setups, such as continuous media perfusion to refresh nutrients and remove waste [67,68], and controlled oxygen supply into the droplets [69]. However, it is important to note that these solutions are not universally applicable and depend on the specific design of the microfluidic chip setup. Consequently, when aiming at long-term bacterial cultivation in droplets, the careful consideration and adaptation of these strategies to suit the particular microfluidic system are necessary.

#### 2.3.2. Simulation of Natural Growth Environments

Droplet-based microfluidics provides a single bacterium with a microenvironment that is closer to its natural environment, with the precise control of the temperature, humidity, gas composition, nutrient concentration, and many other parameters. Gas concentration is one of the most influential environmental variables for microbes, dictating metabolic pathways, growth kinetics, reproduction [73], and functional expression [23]. Conventional bulk cultures offer only coarse control: fast-growing aerobes quickly deplete oxygen, suppressing slow-growing anaerobes [74], and in situ oxygen consumption cannot be monitored in real time [75]. Droplet-based microfluidics overcomes these drawbacks. Each picoliter-scale droplet acts as an oil-encapsulated microreactor whose internal gas concentration can be adjusted independently and monitored with integrated optical or electrochemical sensors [76]. As a result, this isolation eliminates inter-colony competition for gases and allows dynamic read outs of oxygen utilization.

This capability is effectively demonstrated in the work of Watterson, who developed an anaerobic droplet platform (Figure 3B) in which single cells were encapsulated and cultured inside an anoxic chamber maintained with an 86% N_2_/4% H_2_ gas mixture, where the oxygen concentration was strictly controlled to be less than 1 ppm to ensure anaerobic conditions. The homogeneous growth of slow-growing colonies was achieved, after which image analysis and DEP sorting isolated targeted droplets [70]. When applied to fecal microbiota transplant samples, Watterson’s study identified 21 resistant strains that had escaped detection on conventional plate culture, demonstrating its power for rare anaerobe discovery.

Droplet-based microfluidics bridges critical gaps in microbial dark matter research by enabling the precise replication of native environments, thereby facilitating phenotypic observations essential for uncultured taxa. For example, in gut microbiota studies, Yin et al. demonstrated that anaerobic droplet cultivation mimicked intestinal niches, permitting the growth of strictly anaerobic *Lactobacillus* and *Bifidobacterium* species [74]. These bacterial phenotypes were undetectable in aerobic bulk cultures due to oxygen sensitivity. This approach successfully isolated probiotic species previously obscured by competitive dominants in traditional plating. Also, Man et al. investigated groundwater microbial communities using microfluidic droplets amended with soil-derived organic matter and microbial necromass to replicate the native ecological niche of *Candidate Phyla Radiation* (CPR) bacteria [23]. This strategy enabled the targeted enrichment of CPR bacteria-essential components of microbial “dark matter.” Phenotypic assessment demonstrated a 13-fold greater CPR enrichment in droplet cultivation than in bulk cultivation, including rare taxa such as *Candidatus Jorgensenbacteria*. The observed co-enrichment of CPR with putative host taxa (e.g., *Flavobacterium*) confirmed that simulating natural nutrient conditions in droplets reveals episymbiotic phenotypes critical for uncultured lineages.

#### 2.3.3. Monitoring and Analyzing Single-Bacterium Growth

Monitoring bacterial physiology at the single-cell level reveals important insights into cellular heterogeneity and uncovers rare phenotypes or functional traits [8]. Droplet-based microfluidics facilitates this analysis by enabling the high-throughput, compartmentalized tracking of morphology, growth kinetics, division patterns, motility, and responses to external stimuli, including antibiotics [77]. For bacterial growth analysis in droplet systems, fluorescence detection, light scattering, and surface-enhanced Raman spectroscopy (SERS) are routinely employed, as summa-rized in Table 2.

Fluorescence detection monitors bacterial growth and metabolic activity through the fluorescent labeling of bacteria or their metabolites and the detection of fluorescence signal intensity and its dynamic changes [83]. This approach offers high sensitivity and real-time detection capabilities [84]. Ismagilov pioneered a bacteria detection method based on plug-based microfluidic technology and a “stochastic confinement” strategy. This approach isolated individual bacteria within nanoliter droplets, concentrating metabolites to enable rapid, fluorescence-based detection without pre-incubation. Monitoring the fluorescence of alamarBlue indicator, the detection of bacteria was achieved, with the detection time scaling inversely with droplet volume: bacteria in 1 nL droplets were detectable within 2 h. This technology facilitates the detection of bacterial cells in complex matrices, overcoming the time-consuming pre-incubation required by traditional methods [85]. Based on this method, Sabhachandani proposed a droplet microfluidic-based phenotypic antibiotic susceptibility testing (AST) platform for bacteria by encapsulating a single bacterium and antibiotics in picoliter droplets, combined with fluorescence imaging to monitor bacterial quantity and morphological changes (Figure 3C). A droplet docking array immobilizes the droplets, allowing time-lapse fluorescence imaging at 7.5 min intervals. Using green fluorescent protein (GFP) for expressing *E. coli* ATCC 25922 and clinical isolate 6937, the system captured growth inhibition and morphological changes induced by ceftazidime and levofloxacin [71]. However, fluorescent labeling requires genetic modification or exogenous dyes, which may perturb bacterial metabolism and limit applicability to unmodified clinical isolates.

Light scattering provides a label-free alternative by measuring the changes in scattered light intensity caused by growing bacteria. Building on this principle and leveraging its advantages, Pacocha et al. developed a system for bacterial density analysis based on Mie scattering, using optical fibers to detect light signals from nanoliter droplets at 1.2 kHz throughput [72]. This method avoids genetic or chemical labeling and was validated on 12 Gram-positive and Gram-negative bacterial strains, including *E. coli* and *Staphylococcus aureus* (*S. aureus*) (Figure 3D). It also enabled the determination of antibiotic minimal inhibitory concentration (MIC) and phenotypic heterogeneity at the single-cell level. Due to its broad compatibility with bacteria of varying morphologies and growth characteristics, this technique significantly expands the potential applications of droplet microfluidics in microbiological detection.

Surface-enhanced Raman scattering offers a detailed chemical characterization of bacterial composition, including nucleic acids, proteins, and lipids. This analytical technique produces highly specific and sensitive assessments of bacteria’s structural and molecular features [86]. For example, Huang et al. developed a microfluidic microwell device with integrated SERS (microwell–SERS system). This system employs a triangular microwell structure for bacterial encapsulation, combining centrifugal enrichment with SERS to enable rapid AST for low bacterial concentrations (10^3^ CFU/mL) [87]. Specifically, this methodology capitalizes on the high surface area-to-volume ratio of microwells to increase bacterial concentration efficiency, where centrifugation facilitates bacterial accumulation in the well corners. The subsequent SERS detection of bacterial metabolites supports researchers in discriminating between sensitive and resistant strains within 2 h. The system further enables high-throughput parallel detection through microtiter arrays, providing the possibility of the simultaneous analysis of multiple antibiotic concentrations or multiple strains.

## 3. Applications of Droplet-Based Microfluidics in Single-Bacterium Analysis

### 3.1. Antibiotic Susceptibility Testing

Antimicrobial resistance (AMR) constitutes a global health threat, having caused 1.27 million deaths directly attributable to and 4.95 million deaths associated with AMR in bacteria during 2019 [88]. AST remains a critical tool for guiding targeted antimicrobial therapy by determining the minimum inhibitory concentration (MIC) of antibiotics. However, conventional AST methods rely on bulk culture-based assays that take two to three days and fail to resolve single-cell heterogeneity [89]. In contrast, droplet-based microfluidics dramatically shortens this process to a matter of hours while enabling the simultaneous analysis of tens of thousands of individual microreactors [90]. Consequently, this technology provides an exceptionally powerful platform for rapid, sensitive, high-throughput AST at the single-cell level.

**Figure 4:**
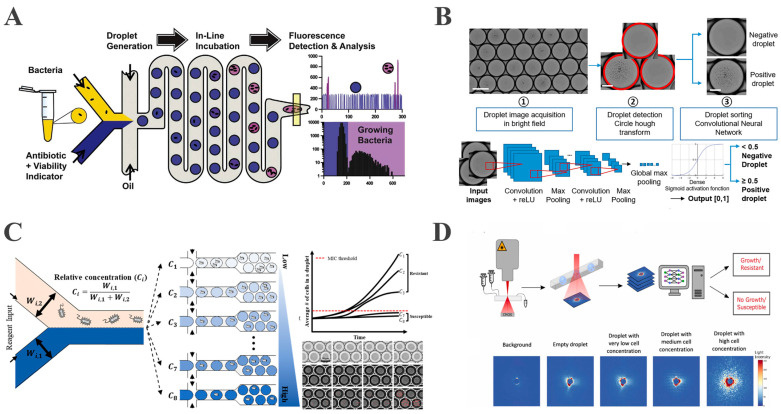
Droplet-based microfluidics enables rapid, high-throughput, and diverse detection strategies for AST. (**A**) A rapidly integrated single-cell biosensing platform for bacterial growth detection and antimicrobial susceptibility assessment (reproduced with permission from [91], copyright 2017, Elsevier). (**B**) A droplet microfluidic platform combined with deep learning for rapid, label-free AST (reproduced with permission from [93], copyright 2024, Elsevier). (**C**) A droplet microfluidic platform combined with deep learning for rapid, label-free antimicrobial susceptibility testing (reproduced with permission from [97], copyright 2024, Royal Society of Chemistry). (**D**) A schematic of the workflow and exemplary ARS images (reproduced with permission from [98], copyright 2024, Elsevier).

Kaushik et al. developed the integrated droplet microfluidic platform dropFAST to monitor real-time bacterial metabolic activity through co-encapsulating single *E. coli*, gentamicin, and the fluorescent indicator resazurin in droplets [91]. Resazurin reduction to fluorescent resorufin provides a quantitative proxy for growth (Figure 4A). The platform integrates droplet generation, incubation, and fluorescence detection and is capable of completing AST within a single hour, corresponding to approximately two to three bacterial replication cycles. Building on prior work, Wang et al. developed an integrated nanodroplet platform for the automated, high-throughput screening of antibiotic combinations. Using pneumatic microvalves to precisely control antibiotic ratios within 50 nL droplets, the platform enabled the pairwise testing of four antibiotics across multiple concentrations. This approach reduced reagent consumption by three orders of magnitude versus conventional methods. Microfluidic tubing facilitated parallel incubation and sequential detection, completing 16 combination screens within 5 h, establishing an efficient tool for synergy analysis [64]. Rosenbaum et al. resolved multi-condition droplet identification through integrated optical encoding and machine learning decoding. By applying pairwise combinations of four fluorescent dyes at six distinct concentrations each, they generated 169 unique optical barcodes for droplet labeling. Machine learning algorithms achieved a decoding accuracy exceeding 99.7%. This system completed AST for 25 distinct conditions within 8 h, enabling the complex parallel screening of multiple antibiotics across varied concentrations, thereby significantly advancing high-throughput capabilities [92]. However, as previously mentioned, a reliance on fluorescent labeling may perturb bacterial physiology, and resazurin is unsuitable for anaerobic bacteria since it serves as an indicator of respiratory activity, thereby limiting the method’s applicability to anaerobic bacteria.

The DropDeepL AST method developed by Riti et al. further optimizes the detection strategy by integrating label-free bright-field imaging with deep learning algorithms [93]. The platform encapsulates bacteria and antibiotics into droplets and utilizes convolutional neural networks (CNNs) to automatically detect morphological alterations. Additionally, this system differentiates between bacterial-proliferating droplets and sterile/growth-inhibited droplets via deep learning algorithms, enabling AST without fluorescent labeling (Figure 4B). In contrast to fluorescent labeling-dependent methods, this approach eliminates labeling-induced perturbations via label-free detection and automates the analysis through the integration of bright-field microscopy with deep learning, achieving complete concordance with broth microdilution (BMD) results across 21 clinical isolates.

Conventional AST encounters further limitations related to throughput due to having to replicate testing at multiple antibiotic concentrations, leading to quantitative AST outcomes constrained by inefficient concentration screening [94,95]. Furthermore, the absence of integrated concentration gradient generation and rapid analytical capabilities impedes the acceleration of clinical AST result acquisition [96].

To address these challenges, Kim et al. integrated a concentration gradient generation module into the droplet platform. This platform employs microfluidic channel width ratios to precisely control the mixing ratios between antibiotics and bacterial suspensions, constructing an antibiotic concentration gradient network that simultaneously generates droplets containing eight antibiotic concentrations with a single bacterium [97]. By integrating label-free phase contrast microscopy with automated cell counting algorithms, multi-concentration MIC determination was accomplished within 3 h (Figure 4C).

The ARS-CNN platform developed by Graf et al. further augmented AST’s capabilities through the deployment of 2D angle-resolved scattering (ARS) sensors to record laser scattering patterns from bacteria within droplets [98]. Upon the laser irradiation of droplets traversing microfluidic channels, bacterial proliferation drives measurable alterations in scattered light intensity and angular distribution, factors which directly correlate with bacterial concentration and cellular morphology (Figure 4D). This platform thus enables real-time predictions of dynamic bacterial concentration changes via neural network analysis of scattering images, processing tens of thousands of droplets within minutes while reducing AST time to 1–2 h. Furthermore, it quantifies single-cell heterogeneity based on ARS patterns, with the single-cell identification of resistant subpopulations providing direct insights into resistance mechanisms and overcoming limitations in heterogeneity detection faced by conventional population-based methods.

### 3.2. Enzyme Screening and Directed Evolution

Enzymes function as essential biocatalysts in synthetic biology, industrial catalysis, and biomedicine. Nevertheless, natural enzymes frequently entail various limitations, including insufficient catalytic activity, narrow substrate specificity, and low stability, hindering their ability to fulfill requirements in complex applications [99,100]. To address such limitations, directed evolution systematically optimizes enzymatic catalytic efficiency, substrate scope, and stability through mimicking natural selection integrated with high-throughput screening, serving as a pivotal methodology for modifying natural enzymes and developing artificial enzymes [101,102].

**Figure 5:**
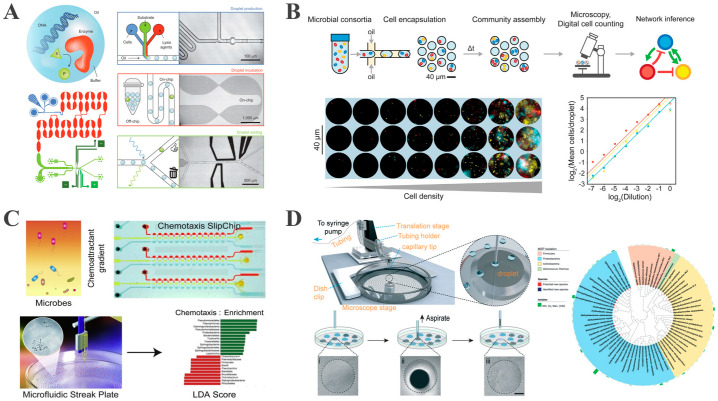
Droplet-based microfluidics for representative applications in single-bacterium analysis. (**A**) Microfluidic platform for the directed evolution of aldolases (reproduced with permission from [104], copyright 2016, Springer Nature). (**B**) Overview and characterization of microbial interaction network inference in a microdroplet (reproduced with permission from [105], copyright 2019, Elsevier). (**C**) Chemotactic screening of imidazolinone-degrading bacteria by microfluidic SlipChip (reproduced with permission from [106], copyright 2019, Elsevier). (**D**) Development of an improved high-throughput MSP platform to accelerate microbial isolation from deep sea sediments (reproduced with permission from [107], copyright 2020, Royal Society of Chemistry).

Critically, droplet-based microfluidics has revolutionized enzyme screening and directed evolution. Compared with traditional methods (e.g., 96-well plates), which are limited to 10^3^–10^4^ variants per day, droplet-based microfluidics permits ultrahigh-throughput screening at 10^7^–10^8^ variants/day [103]. For example, Obexer et al. demonstrated a bacterial fluorescence-activated droplet sorting (FADS) platform that co-encapsulates single *E. coli* cells expressing enzyme variants with fluorescent substrates in picoliter-scale droplets. Sorting at 2000 droplets per second (Figure 5A) produced aldolase variants displaying a 30-fold additional improvement over a pre-optimized scaffold, culminating in a 10^9^-fold rate acceleration and achieving a performance comparable to natural class I aldolases [104]. The engineered enzyme retained high stereoselectivity and accepted diverse substrates, underscoring the power of droplet FADS for fine-tuning complex, multi-step reaction mechanisms.

Furthermore, Zurek et al. addressed detection limits by expanding single bacterial cells into monoclonal microcolonies inside droplets [69]. Continuous oxygenation in a microfluidic reactor grew each encapsulated single cell to 20–30 identical clones. Subsequent “pico-fusion” precisely injected substrate and lysis buffer, increasing the reaction signal 12-fold and lowering the detection limit from 1300 to approximately 100 molecules per enzyme. This strategy markedly enhances both sensitivity and screening accuracy, offering a broadly applicable paradigm for directed evolution campaigns.

### 3.3. Microbial Cell–Cell Interaction: Droplet-Based Co-Culture System

Microbial communities, widely distributed in soil, aquatic, and gastrointestinal environments, are structured and functionally driven by intricate interspecies interactions, such as symbiosis, competition, and predation [108]. Studying these interactions within native-like environments is essential for understanding microbial ecology and metabolic dynamics. Co-culture systems offer a powerful means of reconstructing natural microbial networks, revealing previously unknown metabolic dependencies, accelerating the discovery of functional strains, and enabling the study of uncultured microorganisms by simplifying community composition [109,110]. As such, microbial co-culture represents a robust approach for analyzing intercellular interactions and accessing previously inaccessible microbial taxa.

Droplet-based microfluidics has emerged as a superior alternative to conventional co-culture methods, offering greater precision, scalability, and throughput. This technology enables the parallel generation of tens of thousands of picoliter-scale microreactors, allowing for the capture of rare microbial events while avoiding population-level averaging effects [111].

To advance this technique, Hsu et al. developed the Microbial Interaction Network Inference in microdroplets (MINI-Drop) platform. This system stochastically encapsulates mixed microbial flora into microdroplets using a droplet-based co-culture system, integrated with fluorescent labeling and computer vision techniques, to quantify the absolute abundance of each species within individual droplets (Figure 5B). By comparing cell counts between monoculture and co-cultured droplets, MINI-Drop allows the inference of both the type (positive or negative) and strength of microbial interactions, as well as the modulation of interaction networks under different environmental conditions, including tri-strain communities [105].

Additionally, Terekhov et al. established an ultrahigh-throughput double emulsion droplet platform for screening microbial interactions. In their application, oral microorganisms from Siberian bear were co-encapsulated with fluorescently labeled *S. aureus* within water-in-oil-in-water (W/O/W) droplets. Using flow cytometry at rates up to 30,000 droplets per second, droplets exhibiting diminished *S. aureus* fluorescence, indicating antagonistic activity, were rapidly identified. This platform enabled the direct isolation of *Bacillus* strains with antimicrobial activity from the native microbiome, offering a novel high-throughput approach for antibiotic screening and microbial interaction analysis within complex communities [112].

### 3.4. Cultivation of Uncultivated Microorganisms

Microorganisms are ubiquitous in nature and play critical roles in maintaining the planet’s ecological balance. However, owing to limitations in isolation techniques, only a small fraction of microorganisms has been successfully cultured to date [113]. For example, conventional methods can cultivate approximately a mere 1% of soil microorganisms [114]. Droplet-based microfluidics circumvents this limitation by physically confining a single bacterium in picoliter-to-nanoliter reactors. Isolation prevents dominant bacteria from monopolizing nutrients and provides survival space for slow-growing, rare bacteria [20,115]. High-throughput droplet generation further enables the parallel interrogation of millions of individual cells, significantly enhancing recovery rates for previously uncultured microorganisms [116].

The microfluidic streak plate (MSP) encapsulates single cells in nanoliter droplets that are arrayed at high density on standard Petri dishes, sidestepping the limitations of classical streak plating. This system is specifically designed for high-throughput studies of uncultured or rare environmental microorganisms [117]. Jiang et al. established MSP in three sequential steps: (1) the formation of nanoliter to picoliter droplets by microfluidic emulsification, (2) the stochastic encapsulation of individual cells, (3) the manual or automated transfer of droplets onto oil-covered Petri dishes to establish geometrically ordered, monodisperse arrays [76]. Physical isolation eliminates inter-species competition and nutrient monopolization. Using MSP, Jiang et al. isolated four *Mycobacterium* strains capable of degrading polycyclic aromatic hydrocarbons (PAHs) and discovered a novel fluorescent, anthracene-degrading bacterium, demonstrating MSP’s potential for uncultured microbial studies.

Building upon Jian et al.’s work, Chen et al. developed an integrated “chemotaxis-driven sorting-droplet culture” process using MSP technology, coupled with a microfluidic SlipChip for chemotaxis screening [106]. This approach employs SlipChip to establish chemical gradients (e.g., imidazolinone herbicides), inducing chemotactic bacterial migration toward the gradient source and thus enriching the target degraders, followed by single-cell cultivation via MSP (Figure 5C). Using this integrated approach, Chen et al. achieved the selective enrichment of the targeted functional bacteria via chemotaxis screening, which significantly enhanced target strain isolation efficiency and increased imidazolinone degradation efficiency by approximately 10% compared with traditional enrichment methods. Furthermore, the rare bacterial genus *Ochrobactrum* was identified as playing a critical role in degradation, offering a novel screening strategy for efficient pollutant-degrading microorganisms.

Hu et al. further optimized the MSP technique by developing a semi-automated droplet picker and a long-lasting humidity control device to facilitate the isolation of rare microorganisms from deep sea sediments [107]. This technology involves generating picoliter droplets via a modified microfluidic device, followed by the automated scribing of spiral trajectories and the precise extraction of target droplets using a 3D-printed microscope-guided droplet picker. A sealed incubation chamber maintained at 98% relative humidity enables low-nutrient cultivation for up to five months (Figure 5D). This method offers several advantages over traditional approaches. Firstly, the integration of a robotic arm with a microscope-based picker tripled droplet picking efficiency while eliminating the risk of manual contamination. Secondly, stable humidity control created favorable conditions for the growth of slow-growing deep sea bacteria. With this approach, Hu et al. successfully isolated 772 bacterial strains, including 15 represented novel species.

With these innovations, the MSP platform substantially outperforms traditional plate-based methods in both species diversity and the ability to recover rare microbial taxa, highlighting its strong potential for uncovering novel microbial resources across diverse environments.

## 4. Challenges and Prospects

Droplet-based microfluidics offers unique advantages such as single-cell resolution, a high throughput, miniaturization, a high resolution, and automation, making it a powerful tool for microbial culture, screening, and monitoring. However, several key challenges still limit its broader application in single-bacterium analysis.

One major challenge is the complexity of system integration and operation. Most droplet generation systems rely on external driving devices, such as syringe pumps, to drive fluid flow. For example, traditional T-junction or flow-focusing structures depend on precisely regulated two-phase flow rates to generate monodisperse droplets. This dependence on external power sources limits portability and restricts the use of microfluidic systems in point-of-care diagnostics and field-based studies. Another limitation is the absence of fully integrated systems that combine droplet generation, bacterial culture, and functional analysis within a single device. In many studies, droplet generation is separated from culture and detection steps. For instance, in fluorescence-activated droplet sorting, bacteria are first encapsulated in droplets, then incubated off-chip, and finally reinjected into a detection module for analysis. This segmented workflow increases the risk of contamination and makes it difficult to conduct real-time monitoring of bacterial dynamics. A further technical challenge lies in the real-time regulation and monitoring of key physicochemical parameters inside droplets. Critical factors, including pH and osmotic pressure, remain difficult to control and measure accurately. These limitations hinder long-term investigations into microbial growth, metabolic activity, and environmental responsiveness at the single-cell level.

Notably, advancements in pumpless actuation, integrated microfluidic platforms, and machine learning techniques have enabled novel approaches for single-bacterium analysis. Pumpless actuation eliminates the reliance on external power sources (e.g., syringe pumps), making it particularly suitable for field applications and resource-limited settings, it thus represents a crucial approach for achieving rapid on-site diagnostics. For instance, digital microfluidic systems manipulate droplets across electrode arrays via electrowetting, facilitating pumpless droplet sorting and reagent mixing, thereby offering a compact platform for in situ microbial analysis in extreme environments. Functionally integrated microfluidic devices, meanwhile, can be engineered to enable full-process automation, incorporating droplet generation, incubation, and detection modules within a single integrated platform, effectively mitigating contamination risks and overcoming the dynamic monitoring limitations associated with conventional multi-step procedures. Lastly, machine learning algorithms facilitate the advancement of intelligent droplet microfluidics by permitting real-time image analysis and dynamic modeling. For instance, deep learning algorithms enable the automated tracking of bacterial division cycles and morphological alterations within microfluidic droplets while simultaneously characterizing heterogeneous antibiotic responses. When integrated with neural network algorithms in particular, FADS predicts bacterial metabolic states through real-time fluorescence signal analysis, enabling the precise screening of the targeted strains.

## 5. Conclusions

This review comprehensively summarized recent advances in droplet-based microfluidic technologies for single-bacterium studies, highlighting their distinctive capabilities in single-cell encapsulation, isolation, cultivation, and functional characterization. This review has summarized the principles and implementations of droplet generation, sorting, and microbial in situ culture, with a particular focus on their applications in antibiotic susceptibility testing, enzyme-directed evolution, microbial interaction analysis, and the high-throughput isolation of unculturable microorganisms.

By leveraging the physical isolation characteristics of picoliter-scale droplets, droplet-based microfluidics effectively overcomes several inherent limitations of traditional microbiological methods, including the loss of cellular heterogeneity due to population averaging, the difficulty in screening slow-growing bacteria, and the unculturable microbial “dark matter”.

While promising, this technology still faces challenges, including the reliance on external actuators for droplet generation, potential contamination introduced by multi-module operations, and the limited ability to precisely regulate the microenvironment in real time. Nevertheless, ongoing innovations in micro–nano manufacturing, real-time sensing, and machine learning algorithms are rapidly driving the development of fully integrated, intelligent, and label-free platforms. These systems will enable unprecedented control over synthetic microbial ecosystems. For instance, by creating programmable microenvironments with tailored gradients of nutrients and signaling molecules, researchers are able to engineer bacterial consortia for biomanufacturing. Additionally, these platforms will help to decode complex microbial interactions—such as resource sharing, competition, and antagonism through the high-throughput co-cultivation of environmental consortia within spatially segregated microdroplets. Furthermore, convergence with single-cell multi-omics (e.g., genomics, transcriptomics, metabolomics) will bridge phenotypic observations with molecular mechanisms. This integration empowers the deconvolution of genotype–phenotype relationships in rare uncultured taxa, transforming our understanding of microbial functional potential and adaptive evolution across clinical, industrial, and environmental contexts. These advancements promise broader applications, such as rapid clinical diagnostics and microbiome studies in extreme environments. For instance, the integration of droplet-based microfluidics with single-cell sequencing enables the precise detection of rare pathogens and facilitates the genomic tracing of transmission chains, providing robust support for infectious disease surveillance and outbreak control. Similarly, in the context of environmental remediation, droplet-based microfluidics offers an efficient strategy for isolating microbial strains capable of withstanding heavy metal stress or degrading persistent organic pollutants (POPs) from extreme environmental samples such as polar permafrost and deep sea sediments.

## Figures and Tables

**Table 1:** Methods and related applications of droplet-based microfluidic manipulation modules.

Manipulation	Technical Approaches	Applications
Generation	Generate monodisperse droplets through the interaction of continuous and dispersed phases	Encapsulation of single bacterium using droplets [24,25]
Splitting	Transform a single parent droplet into multiple sub-droplets	In-droplet cell concentration [26], single-cell protein profiling [27]
Merging	Merge multiple droplets into a single droplet	Single-cell pairing [28], targeted lysis of bacterial host cells [29], targeted single-cell RNA and DNA sequencing [30], drug discovery [31], genomic analysis [22], response to antibiotics [32]
Sorting	Pick out target droplets from a large number of droplets	Selection of single cell [33] and enzyme [34], single-cell transcriptomic analysis [35]
Incubation	Incubate droplets under suitable temperature and gas conditions	Analysis of single cell, DNA [36], and AST [37]

**Table 2:** Detection techniques for bacterial single-cell analysis in droplet-based microfluidics.

Detection Techniques	Strengths	Limitations	Applications
Fluorescence	High sensitivity, multiplexing capability, high resolution	Labeling required, photobleaching	Gene expression [78], antibiotic susceptibility testing [71]
Light scattering	Label-free, low cost	Low sensitivity for small bacteria, poor specificity	Bacterial density monitoring [72], single-cell sequencing [79]
SERS	Ultrahigh sensitivity, photostability, label-free operation	Nanoparticle reproducibility, complex substrate design	Single-cell cytokine analysis [80], bacterial screening [81]
Electrical detection	Fast speed, real-time analysis	Signal interference from media, sensor fouling	Single-bacterium physiological status measuring [82]

## Data Availability

No new data were generated or analyzed in support of this research.
